# On the Acquisition of Polarity Items: 11- to 12-Year-Olds' Comprehension of German NPIs and PPIs

**DOI:** 10.1007/s10936-021-09801-3

**Published:** 2021-08-23

**Authors:** Juliane Schwab, Mingya Liu, Jutta L. Mueller

**Affiliations:** 1 Institute of Cognitive Science, Osnabrück University, Wachsbleiche 27, 49090 Osnabrück, Germany; 2 Department of English and American Studies, Humboldt University of Berlin, Berlin, Germany; 3 Department of Linguistics, University of Vienna, Vienna, Austria

**Keywords:** Negation, Polarity items, Language acquisition, Sentence comprehension, Judgment task, Corpus study

## Abstract

Existing work on the acquisition of polarity-sensitive expressions (PSIs) suggests that children show an early sensitivity to the restricted distribution of negative polarity items (NPIs), but may be delayed in the acquisition of positive polarity items (PPIs). However, past studies primarily targeted PSIs that are highly frequent in children’s language input. In this paper, we report an experimental investigation on children’s comprehension of two NPIs and two PPIs in German. Based on corpus data indicating that the four tested PSIs are present in child-directed speech but rare in young children’s utterances, we conducted an auditory rating task with adults and 11- to 12-year-old children. The results demonstrate that, even at 11–12 years of age, children do not yet show a completely target-like comprehension of the investigated PSIs. While they are adult-like in their responses to one of the tested NPIs, their responses did not demonstrate a categorical distinction between licensed and unlicensed PSI uses for the other tested expressions. The effect was led by a higher acceptance of sentences containing unlicensed PSIs, indicating a lack of awareness for their distributional restrictions. The results of our study pose new questions for the developmental time scale of the acquisition of polarity items.

## Introduction

Polarity-sensitive expressions (PSIs) are words or multi-word expressions that are limited in their distribution to a range of so-called licensing environments (Chierchia 2004, 2013; Giannakidou 1998, 2019; Israel 1996, 2011; Krifka 1995; Ladusaw 1979; Szabolcsi 2004; *among others*). We distinguish between negative polarity items (NPIs) like *ever*, which require a negative context to be licensed (1a), and positive polarity items (PPIs) like *already*, which are anti-licensed^1^ by negative contexts (1b). Within and across languages, there is a large number of words that are polarity-sensitive, with substantial variation in their lexical categories: PSIs in English, for instance, include indefinites like *ever, any* (NPIs)*, some* (PPI), degree modifiers like *at all, all that, much* (NPIs), *pretty, somewhat* (PPIs), and idiomatic expressions like *to lift a finger, a red cent* (NPIs), *all the time in the world* (PPI). In turn, the environments that license NPIs and/or anti-license PPIs themselves are extremely varied—in addition to sentential negation as in (1a), NPIs can also be licensed under the scope of negative quantifiers like *no* or *nobody* (2a), under downward-entailing^2^ operators like *few* (3a), in nonveridical^3^ contexts like questions (4a) or the antecedent of conditionals (5a), and in superlatives (6a). Other (so-called *strong*) NPIs, by contrast, are licensed only in the strongest negative environments (e.g., *either*, which is only licensed in at least anti-additive^4^ environments). Similar patterns arise for PPIs: *Already* is acceptable in questions (4b), conditionals (5b) and superlatives (6b), while the PPI *some* is additionally acceptable under downward-entailing operators like *few* (*Few people had eaten something for breakfast*).

**Table:** 

(1) a. Mary has*(**n’t**) *ever* been to Paris. b. John has(#**n’t**) *already* left the party.
(2) a. **Nobody**/*Somebody I know has *ever* been to Paris. b. #**Nobody**/Somebody has *already* left the party.
(3) a. **Few**/*Many of my students have *ever* been to Paris. b. #**Few**/Many people have *already* left the party.
(4) a. Has Mary *ever* been to Paris**?** b. Has John *already* left the party**?**
(5) a. **If** Mary *ever* goes to Paris, she must visit the Louvre. b. **If** John has *already* left the party, I cannot introduce him to Mary tonight.
(6) a.The Louvre is **the best** museum that Mary has *ever* been to. b. The **funniest** person who has *already* left the party is John.
(7) **No one** thinks that John has**n’t** *already* left the party.
(8) A: John *already* left the party. B: No, John HAS**N’T** *already* left, I just saw him.

Finally, the distribution of PPIs is further complicated by the fact that they can be *rescued* (Szabolcsi 2004) if the negation scoping over the PPI is itself outscoped by an (at least) downward-entailing operator (7), or if the negation is understood as an emphatic denial or contrast to an earlier assertion (8). By comparison, unlicensed NPIs always make the sentence ungrammatical; NPI and PPI violations are thus qualitatively distinct (Liu and Iordǎchioaia 2018; see also Liu et al. 2019 for experimental evidence).

Relatedly, deriving a general licensing property from the distributional facts outlined above has been a challenging pursuit. To highlight just a few controversial issues, let us consider two main approaches to polarity sensitivity: First off, scalar approaches (Israel 1996, 2011; Kadmon and Landman 1993; Krifka 1995*; among others*) hold that PSIs are expressions whose usage is restricted to contexts that license particular scalar inferences.^5^ For the NPI *ever*, for instance, the assertion with the NPI has to be more informative than its alternatives to license its use. This is straightforwardly assured through the entailment relations between the former and the latter in contexts that are (at least) downward entailing. For questions (4), conditionals (5), or superlatives (6), however, it requires reliance on weaker concepts of entailment (e.g., Strawson-entailment, von Fintel 1999) or on a different notion of informativity (van Rooy 2003). Alternatively, the veridicality-based theory by Giannakidou (1998, 2019) holds that PSIs are sensitive to the veridicality of the environment, such that NPIs like *ever* are restricted to nonveridical contexts, whereas PPIs like *already* are repelled by contexts that are antiveridical, i.e., contexts where the falsehood of the proposition is entailed or presupposed. However, this approach, too, faces challenges, one of which is that one has to appeal to theoretically unattractive *rescuing* operations for NPIs that appear in contexts that do entail the truth of the proposition (e.g., *Only Mary has ever been to Paris*).

All in all, the intricacies of PSI licensing—as exemplified through their distributional differences and the difficulty to generalise to a unifying licensing property—constitute a challenge for language learners: Given that PSIs vary in strength, and may not occur equally frequently with their full set of licensing environments,^6^ it is an open question how and at what point in development children learn to generalise from the limited input of a (licensed) PSI to an unconscious knowledge of all the contexts that can or cannot license said expression. To our knowledge, existing studies on the acquisition of PSIs have largely targeted the comprehension and production of highly frequent PSIs like *any* (O’Leary and Crain 1994; Tieu 2013; Tieu and Lidz 2016), *some* (O’Leary and Crain 1994), or the Dutch NPI *hoeven* (‘need’) (Lin et al. 2015, 2018), in relatively young age groups. The findings, summarised in more detail below, are that 5-year-olds are almost adult-like in their production of the tested NPIs, whereas the data is not as clear-cut for the PPI *some*. The present study will complement this work through an investigation of 11–12-year-olds’ comprehension of German NPIs and PPIs. Since children’s language faculty is still undergoing substantial development throughout childhood and adolescence, with important maturational milestones at age 10–11 (see below), our study provides important new insight on the comprehension of PSIs in a more mature, yet developing, language system. We identified four PSIs that are present, but infrequent, in German child language corpora, and used an auditory naturalness rating task to assess whether children at age 11–12 are aware of the distributional restrictions of the tested PSIs. The results show the correct directionality in children’s responses, i.e., a dispreference for un-/anti-licensed PSIs, but only for one of the tested expressions, the NPI *jemals* (‘ever’), did we find the same clear-cut distinction into grammatical and ungrammatical uses as for adults. For the three other expressions, children did not categorically reject un-/anti-licensed uses.

## Previous Studies on the Acquisition of PSIs

The literature on the acquisition of PSIs primarily centres around the English NPI *any* and the Dutch NPI *hoeven* (‘need’). For *any*, Tieu (2013) reports that children are remarkably consistent in producing *any* within the scope of a licenser (primarily under the scope of sentential negation, Tieu 2013:53) in spontaneous speech, using *any* in affirmative contexts in only 2% of the analysed transcripts. This finding is consistent with earlier results from an elicited production study by O’Leary and Crain (1994) (reported in Gualmini 2004): 4–5-year-old children were prompted with short scenarios intended to provoke the use of *some* or *any* in their response. After a brief story, a puppet uttered a false statement about what had happened as in (9a) and (10a). Children were prompted for a response by asking ‘*What really happened?*’. Despite the use of *any* in the puppet’s utterance in (9a), children would usually respond with affirmative utterances using *some* (e.g., *Every dog got some food*) rather than *any* (*Every dog got any food). Together, the corpus and experimental data suggest that children show an early sensitivity to the distributional restriction of *any* in their produced speech. At the same time, it does not constitute direct evidence that children would actually reject *any* in all positive contexts, that is, that they have the grammatical knowledge that *any* is incompatible with positive linguistic environments.

**Table:** 

(9) *Context*: The dogs are hungry, and every dog ate something. a. *Puppet*: Only one dog got any food. b. *Experimenter*: What really happened?
(10) *Context*: The dogs are hungry, but one of the dogs did not eat anything. a. *Puppet*: Every dog got some food. b. *Experimenter:* What really happened?

Mirroring the English data, spontaneous speech transcripts for Dutch *hoeven* (‘need’), too, indicate that children only rarely produce unlicensed instances (4% of the data were classified as non-adult-like) (Lin et al. 2015). Moreover, across all analysed transcripts, sentential negation was the most frequent licenser (95.9% in children’s utterances) and only at age 4–5 did negative quantifiers emerge as a second licenser used by children. Using an elicited imitation task, Lin et al. (2018) further showed that the set of licensers used by children widens over time: 2–5-year-olds would listen to sentences containing *hoeven* licensed by *niet* (‘not’), *geen* (‘no’), *niemand* (‘nobody’), *weinig* (‘few’), *alleen* (‘only’), or without a licenser, and were told to repeat the sentence as accurately as possible. Non-repetitions or changes in the repeated sentence are assumed to indicate that the stimulus was inconsistent with the child’s grammar (Lin et al. 2018:53). The authors found age-related differences in the repetition rates: *Niet* (‘not’) and *geen* (‘no’) emerged as licensers before the age of three, whereas the repetition rate for *niemand* (‘nobody’), *weinig* (‘few’), and *alleen* (‘only’) only increased at around 4 years of age. Moreover, children only rarely changed a sentence containing licensed *hoeven* to a sentence in which *hoeven* was not licensed. In response to sentences containing unlicensed *hoeven*, however, children changed the sentence in more than half of the responses, i.e., they added a licenser or substituted unlicensed *hoeven* with a different word in order to make the sentence grammatical.

From the Dutch data, Lin et al. (2015, 2018) argue for an input-driven conservative widening strategy, wherein children’s acquisition of *hoeven* is marked by two distinct developmental stages: First, a stage of analysing *hoeven* as being in a lexical dependency with the sentential negator *niet* or the negative quantifier *geen*, which is falsified by language input where *hoeven* receives other licensers. Then, a reanalysis to associate *hoeven* with an abstract NEG (negation) operator that encompasses the full range of licensing environments. This account provides a possible developmental pathway for the acquisition of NPIs that explains how children generalise from limited input to the full set of licensers and that accounts for the rarity of unlicensed NPIs in children’s speech. However, it remains unclear whether this approach is directly applicable to other NPIs: *Hoeven* is quite particular in so far as the corpus data indicates that it overwhelmingly appears with *niet* (80.8%) or *geen* (15.4%) as licensers in child-directed speech (Lin et al. 2015). A conservative widening strategy seems less plausible for NPIs that consistently appear with a greater variety of licensers, making the initial analysis step of lexically associating such NPIs with a particular lexical licenser much less attractive.

With regard to the acquisition of PPIs, we are only aware of a handful of studies. Within the same elicited production study reported above, O’Leary and Crain (1994) tested for children’s production of sentences containing the PPI *some*. After hearing the context in (10), children produced more negative utterances containing the NPI *any* (e.g., *No, this dog didn’t get any food*) than the PPI *some* (#*No, this dog didn’t get some food*). However, the results for the PPI *some* were not as clear-cut as for the NPI *any*, which was almost never used in positive contexts. Musolino (1999) further investigated this pattern using a Truth Value Judgment Task (TVJT) with 3–6-year-old children. Children were first presented with a story acted out by puppets (11), concluding with the puppet’s statement in (11a). (11a) is acceptable if the PPI is interpreted as taking wide scope over the negation (*There is someone that the detective did not find*), but is unacceptable under the narrow scope interpretation (*There isn’t #someone/anyone that the detective found*). Although the adult control group consistently accepted the utterance, children rejected it about half of the time, arguing that it was false because the detective found one of his friends. This indicates that children assigned the narrow scope reading to the sentence although this reading is not available in the adult grammar system. Similar findings have also been observed in a TVJT by Xiang et al. (2006).

**Table:** 

(11) *Context:* A detective is playing hide and seek with his two friends. At first, he doesn’t find any of them, but eventually he discovers one of them behind a tree. He does not find the second friend.
a. *Puppet*: The detective didn’t find someone.

Overall, the results from both tasks suggest that children below the age of six sometimes accept *some* under the scope of negation, and thus do not seem to have mastered its distributional restrictions yet. However, more research on other PPIs and on other languages will be needed to establish whether the acquisition of PPIs is delayed compared to the acquisition of NPIs *in general*, or whether the findings reported here are specific to the contrast between *some* and *any*.

To summarise, the reported research on the acquisition of PSIs focused on just a few (highly frequent) expressions, for which the consensus is that NPIs are consistently used with a licenser from early on, but that the range of licensing expression that a child employs may change, i.e., broaden, over time. By the age of 5, children behaved largely target-like in experimental tasks requiring the production of *any* or *hoeven*. However, since all of the studies tested children who are too young to participate in metalinguistic tasks like grammaticality judgments, we do not have direct evidence for children’s receptive knowledge of the ungrammaticality of NPIs in affirmative contexts. Instead, this conclusion is only available to us by inference from the absence of such constructions in children’s speech. Turning to PPIs, existing research focused on *some*, for which a target-like knowledge of the distributional constraint does not appear to be reached even by the age of six. A delay in the acquisition of PPIs may well be expected given that children cannot infer their distributional constraints from a co-occurrence with other linguistic elements. While NPIs usually appear with a negative element, PPIs do not have overt lexical associates. Instead, children must somehow realise that it is the absence of negation that licenses them, a process made all the more difficult by the presence of exceptions to this rule, such as the occurrence of PPIs under two negative operators or under emphatic denials (see Introduction).

## The Current Study

In order to provide more direct evidence for children’s knowledge of the distributional restrictions of PSIs, we conducted a graded naturalness rating study using NPIs and PPIs in licensing and non-licensing (or anti-licensing) contexts in adults and 11–12-year-old children. This paradigm is a simple but sensitive measure suitable for older children, which allows us to assess immediately whether they perceive PSIs in non-licensing environments as ill-formed rather than having to infer so indirectly from utterances they do (or do not) produce. Following Ambridge (2012) and Karanth and Suchitra (1993), we assume that children can engage with and respond to judgment tasks in the intended manner by the age of 6–8.

Besides the suitability of the task, the more fundamental reason to test the age group between 11 and 12 is that cognitive developmental research indicates that the language faculty is still undergoing significant maturation up until at least age 10–11 (Broce et al. 2015; Nuñez et al. 2011; Skeide et al. 2014, 2016; Vissiennon et al. 2017; Wassenberg et al. 2008; for a review see Skeide & Friederici 2016) and that syntactic and semantic development continues throughout adolescence (Hahne et al. 2004; Schneider and Maguire 2019; Schneider et al. 2016). Neurophysiologically, these maturational processes are apparent both structurally and functionally: Diffusion tensor imaging indicates that the left arcuate fasciculus (AF), a white matter tract that connects frontal with inferior parietal and temporal language areas that is known to be involved in speech and syntax processing, undergoes significant microstructural changes in 5–8 year-olds (Broce et al. 2015). The bilateral AF microstructure was also able to predict performance on receptive and expressive language tests, underscoring that its maturation may play a crucial role in language development. Skeide et al. (2016) confirmed these microstructural changes of the AF in 3–10-year-olds and found that the fibre tract maturation together with activation levels of two language areas connected by the AF (the posterior super temporal gyrus (pSTG) and the left inferior temporal gyrus (left IFG)), predicted accuracy and speed of syntactic comprehension. Two fMRI studies on syntactic and semantic comprehension (Nuñez et al. 2011; Skeide et al. 2014) further suggest that syntax and semantics are only fully segregated into separate language modules by the age of 9–10, while for younger age groups the activation patterns for syntactic and semantic processing largely overlap. Lastly, a range of studies suggest that language development continues throughout adolescence: Testing 6–13-year-olds on passive sentences containing semantic or word-category based syntactic violations, Hahne et al. (2004) found that only 13-year-olds and adults showed the early left anterior negativity (ELAN) component that indicates an automatic detection of syntactic violations, whereas younger age groups only showed the later P600 component of syntactic processing. Two recent studies employing combined ERP and time–frequency analyses of EEG data (Schneider and Maguire 2019; Schneider et al. 2016) further show age-related differences in the neural oscillations associated with language processing: Children up to the age of 13 displayed non-adult-like oscillatory (beta and theta band) activity in response to syntactic and semantic violations. Cross-sectional behavioural studies on 5–17-year-olds (Dick et al. 2004) and 5–15-year-olds (Wassenberg et al. 2008) lend additional support to the neurophysiological findings reviewed here: Dick et al. (2004) found that complex language comprehension improved significantly with age, particularly in the comparison between children aged 9 or older compared to 5–8-year-olds, and Wassenberg et al. (2008) found a linear increase in performance up until sixth grade (around 11 years old). The 11–15-year-olds in the Wassenberg et al. study did not differ in performance, but also had not reached adult comprehension levels yet.

Altogether, the neurophysiological and behavioural literature thus suggests that the language system is still undergoing substantial development in middle childhood and adolescence, but appears to reach important maturational milestones such as the segregation of syntax and semantics around age 10. For an interface phenomenon like PSIs, this development may provide a crucial basis for an increasingly adult-like comprehension. By testing 11–12-year-olds in our study, we can therefore tap into children’s comprehension of polarity-sensitive expressions at a time point where the basic neural foundation for the processing of semantics and syntax, here in the form of the relation between PSI and licenser/anti-licenser, is secured, but where we could still expect differences in the efficiency and accuracy of comprehension compared to an adult control group.

### Corpus Data

We decided to conduct a study on children’s comprehension of two German NPIs, *jemals* (‘ever’) and *so recht* (‘really’), and two German PPIs, *durchaus* (‘quite’/‘indeed’) and *absolut* (‘absolutely’). Their frequencies in the syntactically annotated (tagged-T) archive of the German reference corpus DeReKo (Leibniz Institute for the German Language 2020) are 9.341 for *jemals*, 6.688^7^ for *so recht*, 34.100 for *absolut*, and 89.311 for *durchaus*. The two PPIs thus are more frequent than the NPIs. Do note however that *jemals* can also be shortened to *je*, which occurs much more frequently in the corpus (212.431). German *je* is incredibly multi-functional: Besides the temporal use as *ever, je* can be used to indicate reference to each element in a set (*30 Euro je Person,* ‘30 Euro per person’), can function as conjunction (*je früher, desto besser*, ‘the earlier, the better’), or express that something is conditional on something else (*je nachdem*, ‘depending on’). We were therefore unable to determine the frequency of temporal uses of *je* in this data.

The four PSIs were selected based on their classification as NPI/PPI in the German database of distributionally idiosyncratic items (CoDII; Trawiński and Soehn, 2008), and based on our findings in a corpus search conducted via the German CHILDES database (MacWhinney, 2000). All four PSIs appear in child-directed speech within the corpus, but none of them are frequent in children’s own utterances, underscoring the need of experimental data to identify at what point children acquire these PSIs. Specifically, we analysed all four PSIs’ distribution in five German subcorpora of the CHILDES database: The Caroline (Von Stutterheim 1989), Leo (Behrens 2006), Miller (1979), Rigol (2007), and Wagner (1985) corpora. In total, 1381 CHAT files containing data from children between the age of 1;00 and 14;10 were analysed. For *jemals,* we searched both for *je* and for *jemals*, manually determining whether the instances of *je* were temporal. We report the combined number of instances, but indicate in Table 1 which ones were *je* and which ones were *jemals*. Each of the four PSIs occurred with similar frequency in child-directed speech (Table 1) and was always appropriately licensed. Interestingly, the NPI *je(mals)* appears with a very diverse set of licensers, including downward-entailing environments, nonveridical environments (questions and conditionals), but also superlatives and comparatives. While *so recht*, too, can be licensed by some of these contexts, the child-directed speech is clearly biased towards licensing by sentential negation. Critically, recall that under the conservative widening strategy proposed by Lin et al. (2015, 2018), this may lead children to initially analyse *so recht* as lexically associated with the sentential negation *nicht*. For *je(mals)*, on the other hand, the diversity of licensers makes this approach less plausible. So far, however, it is unclear whether this could result in a delay of the acquisition of this NPI (because children may initially not be able to identify a specific lexical associate that licenses *je(mals)*), or might in turn facilitate it (due to a faster generalization to the abstract semantic property licensing *je(mals)*). Including both NPIs in our rating study may thus provide some insight on this question.

**Table 1:** Distribution of the four investigated PSIs in child directed speech in the German CHILDES corpora

	*Nicht* (‘not’)	*Kein* (‘no’)	Question	Other licensing environments	Positive	Total
*je(mals)*	3 (*je*)	0	9 (4 *jemals*, 5 *je*)	6 (2 *jemals*, 4 *je*)^a^	0	18
*so recht*	39	1	0	0	0	40
*absolut*	0	0	0	0	36^b^	36
*durchaus*	0	0	0	0	32	32

^a^These were two instances of licensing by a comparative (1 *jemals*, 1 *je*), two in a superlative structure (1 *jemals*, 1 *je*), one in a conditional antecedent (*je*), and one instance licensed by *kaum *‘barely’ (*je*). ^b^Of these 12 scoping above negation

The PPIs *absolut* and *durchaus* both appear exclusively in their licensed form in child-directed speech. Note, however, that among the instances of *absolut*, the corpus search revealed 12 instances (one third of all instances) in which it scopes above a negation, as in ‘*Das ist absolut kein Problem’* (‘This is absolutely no problem’). For *durchaus*, on the other hand, no instance with wide scope negation was found, although ‘*Das ist durchaus kein Problem*’ (‘This is indeed no problem’) is well-formed as well. Once more, it is an open question whether the common occurrence of a PPI scoping above negation may prevent children from successfully acquiring the knowledge that the expression cannot in turn scope under negation. We will return to both the contrast between *je(mals)* and *so recht*, and the contrast between *durchaus* and *absolut*, in the discussion of our experimental results.

Children’s utterances within the analysed corpora show only very few, if any, spontaneous uses of the investigated PSIs (Table 2). *So recht* occurs most frequently, albeit with 7 of the 9 recorded instances coming from a single child, Leo. On the upside, there are no unlicensed PSI uses either, aside from one instance of *so recht* that could not be clearly classified due to word omissions (Table 2).

**Table 2:** Distribution of the four investigated PSIs in children’s speech in the German CHILDES corpora

	*Nicht* (‘not’)	Positive	Unclear	Total
*je(mals)*	0	0	0	0
*so recht*	9 (3;00.24–12;02)	0	1 (3;09.19)	10
*absolut*	0	1 (3;03.13)	0	1
*durchaus*	0	1 (4;08.11)	0	1

Overall, the data from the German CHILDES corpora thus show that all four PSIs appear with a similar frequency in child-directed speech, but critically differ with regard to the distribution of their licensing environments. The data from children’s utterances, on the other hand, are too limited to draw conclusions on the acquisition of these PSIs, and in particular, do not allow us to conclude anything about children’s knowledge of their distributional restrictions. To measure precisely that, we conducted an auditory rating task in which 11–12-year-olds were confronted with licensed and unlicensed uses of the four PSIs. The experiment is reported in the following section.

### Method

#### Participants

36 adults and 40 11–12-year-olds participated in the study. The adult participants (26 female, mean age = 22, age range: 18–26) were students at Osnabrück University participating for course credits. The participating children (23 female, 22 11-year-olds, 18 12-year-olds) were sixth grade secondary school students. They were reimbursed for their participation with a 10 Euro gift certificate to a local book shop. The parents of the participating children reported no developmental delays, neurological disorders, or language disorders in their child. All participants were monolingual German native speakers with normal or corrected-to-normal vision and normal hearing. The experiment was approved by the ethics committee of Osnabrück University.

#### Materials

We created 32 items in eight conditions such that all items contained one licensed and one unlicensed use of each of the four PSIs. As NPI licenser, respectively PPI anti-licenser, we used a negative quantifier (*kein*, ‘no’) in the object position scoping above the PSI. Alternatively, for the licensed PPI conditions, respectively unlicensed NPI conditions, we used a definite determiner (*der*, ‘the’) in the same position (see an example in (12)). We also created 16 grammatical filler sentences that did not contain a PSI: eight sentences with a relative clause, and eight sentences containing two clauses linked by a concessive discourse connective. The complete list of filler and target items is included in the Appendix.

**Table:** 

(12)	a.	*Lukas	*hat*	***dem***	*Arzt*	*in*	*dem*	*Krankenhaus*	*so*	*recht*	*vertraut.*
		Lukas	has	the	doctor	in	the	hospital	so	really_NPI_	trusted
		‘Lukas has really_NPI_ trusted the doctor in the hospital.’									
	b.	*Lukas*	*hat*	***keinem***	*Arzt*	*in*	*dem*	*Krankenhaus*	*so*	*recht*	*vertraut.*
		Lukas	has	no	doctor	in	the	hospital	so	really_NPI_	trusted
		‘Lukas hasn’t really_NPI_ trusted the doctor in the hospital.’									
	c.	*Lukas	*hat*	***dem***	*Arzt*	*in*	*dem*	*Krankenhaus*	*jemals*	*vertraut.*	
		Lukas	has	the	doctor	in	the	hospital	ever_NPI_	trusted	
		‘Lukas has ever_NPI_ trusted the doctor in the hospital.’									
	d.	*Lukas*	*hat*	***keinem***	*Arzt*	*in*	*dem*	*Krankenhaus*	*jemals*	*vertraut.*	
		Lukas	has	no	doctor	in	the	hospital	ever_NPI_	trusted	
		‘Lukas hasn’t ever_NPI_ trusted the doctor in the hospital.’									
	e.	*Lukas*	*hat*	***dem***	*Arzt*	*in*	*dem*	*Krankenhaus*	*absolut*	*vertraut.*	
		Lukas	has	the	doctor	in	the	hospital	absolutely_PPI_	trusted	
		‘Lukas has absolutely_PPI_ trusted the doctor in the hospital.’									
	f.	*#Lukas*	*hat*	***keinem***	*Arzt*	*in*	*dem*	*Krankenhaus*	*absolut*	*vertraut.*	
		Lukas	has	no	doctor	in	the	hospital	absolutely_PPI_	trusted	
		‘Lukas hasn’t absolutely_PPI_ trusted the doctor in the hospital.’									
	g.	*Lukas*	*hat*	***dem***	*Arzt*	*in*	*dem*	*Krankenhaus*	*durchaus*	*vertraut.*	
		Lukas	has	the	doctor	in	the	hospital	quite_PPI_	trusted	
		‘Lukas has quite_PPI_ trusted the doctor in the hospital.’									
	h.	*#Lukas*	*hat*	***keinem***	*Arzt*	*in*	*dem*	*Krankenhaus*	*durchaus*	*vertraut.*	
		Lukas	has	no	doctor	in	the	hospital	quite_PPI_	trusted	
		‘Lukas hasn’t quite_PPI_ trusted the doctor in the hospital.’									

Filler and target sentences were recorded by a female German native speaker. The audio files were subsequently edited to remove periods of silence at the onset or offset of the recordings and to normalise them to the same volume. Both editing steps were conducted using the audio editing software *Audacity*^8^ (Audacity Team 2019). To ensure that there are no prosodic cues towards a sentence’s grammaticality in the stimuli, we recorded four additional sentences for each target item, in which the (anti-)licensing quantifier was replaced by a nonsense syllable (e.g., *Lukas hat ****fla**** Arzt in dem Krankenhaus [so recht / jemals / absolut / durchaus vertraut.]*, ‘Lukas has **fla** doctor in the hospital [really / ever / absolutely / quite trusted.]’). The bracketed segment was then spliced into the conditions of (12), such that both the un-/anti-licensed and the licensed condition had the same auditory signal at the critical point in the sentence where the PSI occurs.

#### Procedure

Adults and children were tested using the same procedure. We used a naturalness rating task with a 7-point Likert scale to assess whether participants are sensitive to the restricted distribution of the tested PSIs. The experiment was programmed and hosted on Ibex Farm (Drummond 2013). Participants wore headphones throughout the experiment. In each trial, they first listened to a sentence. Once the sentence had finished playing, a comprehension question appeared on the screen asking participants to choose the correct completion of a sentence fragment (e.g., for (12): *Lukas is…(a) in the hospital (b) at a retirement home*). Participants could replay the sentence as often as they wanted. After the comprehension question had been answered, a rating scale appeared in its place asking participants to rate the naturalness of the sentence they had just heard. The scalar endpoints were marked with the labels *natural* (7) and *unnatural* (1). We added smiley faces along the scale to illustrate the response scheme. Once participants clicked on a response, the trial ended and the next trial began. Participants saw 32 experimental trials and 16 filler trials presented in a pseudorandom order such that no more than three experimental trials would appear in immediate sequence and two experimental trials with the same PSI would never appear right after each other. The experiment started with two practice trials. The total experimental duration was approximately 15 min.

#### Data Analysis

We used Bayesian ordinal regression models with a cumulative link function (Bürkner and Vuorre 2019) to analyse the rating data. All analyses were conducted using the *brms* package, version 2.12 (Bürkner 2017) in R, version 4.0 (R Core Team 2019). Before the main analysis, we assessed participants’ response accuracy on the comprehension questions. For both adults and children, all participants had a response accuracy > 95%. We therefore did not exclude any participants from our analysis. For all models, the effect of context (licensing/non-licensing) was treatment coded (0, 1); the PSI comparisons were entered as custom contrasts such that the model included a comparison between the two PPIs and the two NPIs, a comparison between *jemals* and *so recht* (the NPIs), and a comparison between *absolut* and *durchaus* (the PPIs), each entered as sum coded contrasts (0.5, −0.5). All models used the maximal random effects structure, including random by-subject and by-item intercepts and slopes for all effects. When necessary to resolve an interaction, the models were rerun using treatment coding for the PSI comparisons. We used uninformative uniform priors on the fixed effects. For each model, 4 chains were run with 4000 sampling iterations each using a warm-up period of 2000 iterations. We report the posterior parameter estimates together with the 95 percent credible intervals and the posterior probability that the parameter value is bigger/smaller than 0. All data and code are available online (see data availability statement).

### Results

#### Adults

**Fig. 1:**
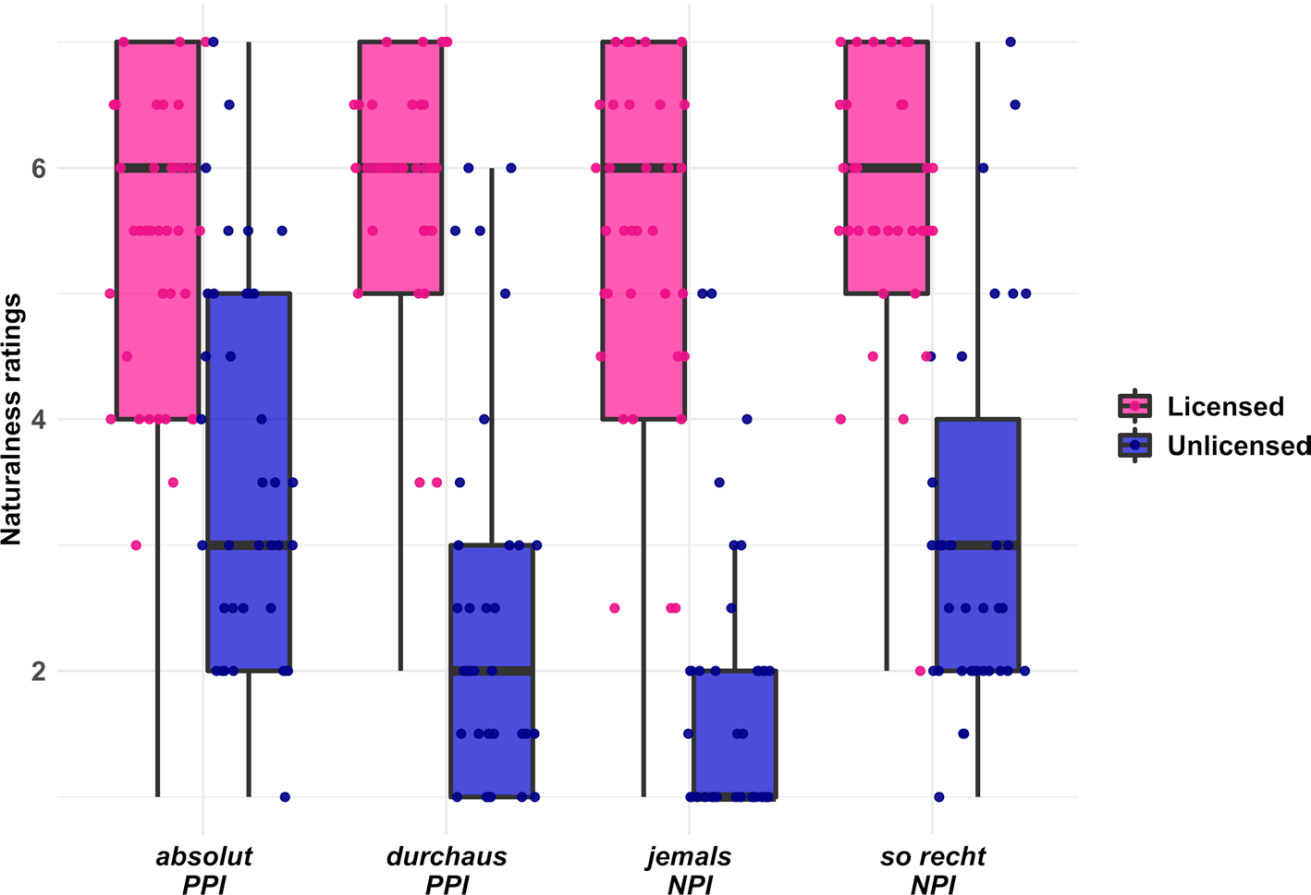
Boxplot of adults’ naturalness ratings for the eight conditions of the experiment. The thick black line shows the median rating per condition, the upper and lower hinges of the box correspond to the first and third quartile. Whiskers extend to the smallest/largest value that is no further than 1.5-times the interquartile range away from the hinges of the box. Individual dots represent each participant’s median rating across the repeated measures

Adults’ rating responses are visualised in Fig. 1. Adult participants demonstrate a clear categorical differentiation between licensed and unlicensed PSI uses: For all tested PSIs, the licensed condition was rated more natural than the unlicensed one (P(β < 0) = 1 for all PSIs). Furthermore, we found an interaction between the licensing status and the contrast between NPIs and PPIs ($\hat{\beta}$ = 0.53, CrI = [0.08, 0.98], P(β > 0) = 0.99)^9^: In licensed conditions, there was no evidence for a difference in the naturalness of the tested NPIs and PPIs ($\hat{\beta}$ = 0.07, CrI = [-0.20, 0.37], P(β > 0) = 0.69), whereas in unlicensed conditions, the model indicated that PPIs were given higher naturalness ratings than NPIs ($\hat{\beta}$ = 0.61, CrI = [0.26, 0.95], P(β > 0) = 1). This is in line with theoretical and experimental work (Liu and Iordǎchioaia, 2018; Liu et al. 2019) that argues that NPI violations and PPI violations are qualitatively distinct, such that NPI violations result in irrescuable ungrammaticality, whereas PPIs in negative contexts can often be saved (see also Introduction). Further comparisons between the tested PSIs demonstrated that both the NPI *so recht* (‘really’) and the PPI *absolut* (‘absolutely’) received somewhat higher naturalness ratings in non-licensing contexts than their counterparts *jemals* (‘ever’) ($\hat{\beta}$ = − 1.33, CrI = [− 1.81, − 0.87], P(β < 0) = 1) and *durchaus* (‘quite’) ($\hat{\beta}$ = − 0.88, CrI = [− 1.29, − 0.46], P(β < 0) = 1). Overall, the difference in the rating for licensed and unlicensed conditions was smaller for *absolut* (‘absolutely’) than for any of the other tested expressions (P(β < 0) = 1 for all comparisons), which is largely because anti-licensed *absolut* was not clearly rejected.

#### 11–12-Year-Olds

**Fig. 2:**
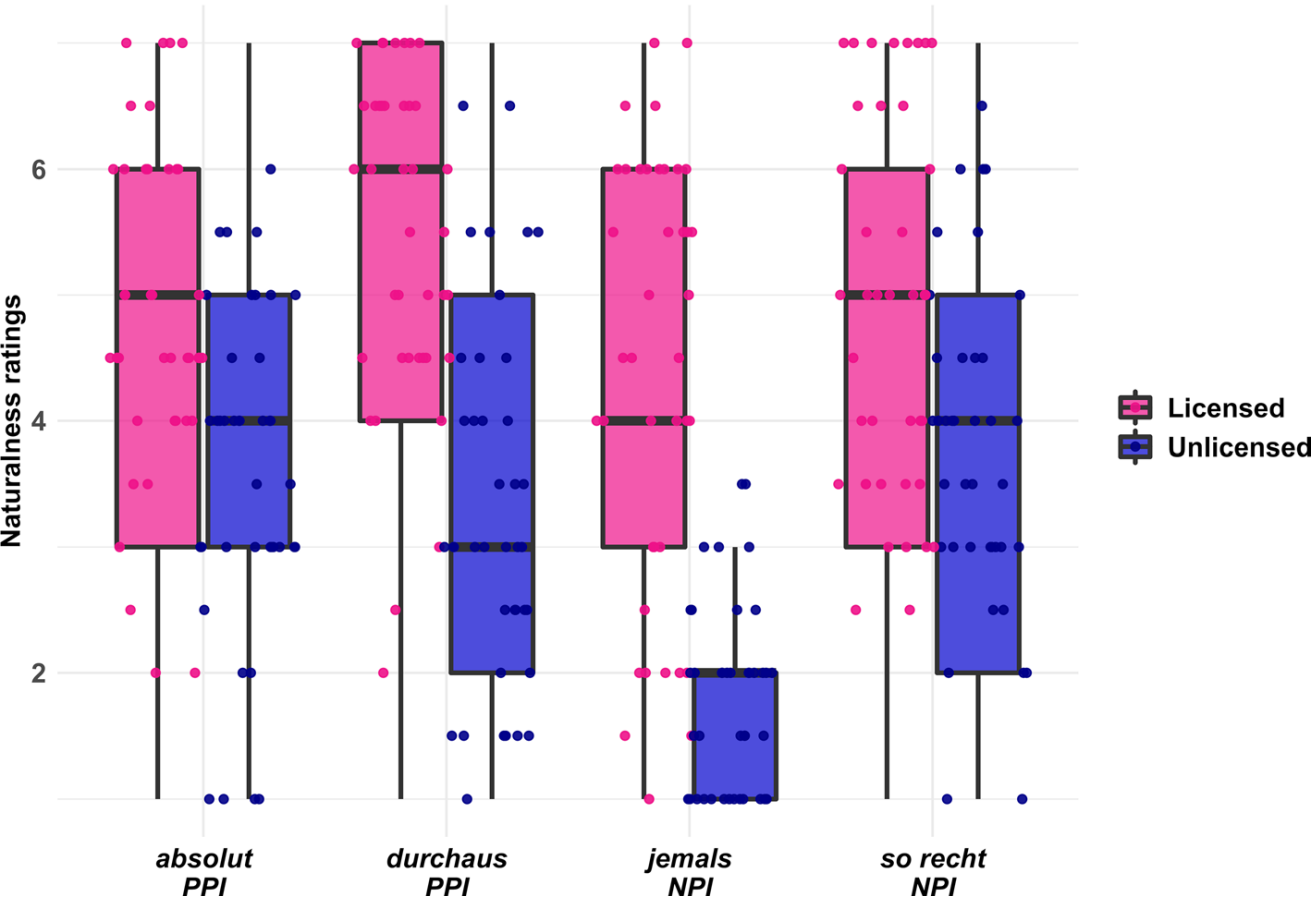
Boxplot of 11–12-year-olds’ naturalness ratings for the eight conditions of the experiment. The thick black line shows the median rating per condition, the upper and lower hinges of the box correspond to the first and third quartile. Whiskers extend to the smallest/largest value that is no further than 1.5-times the interquartile range away from the hinges of the box. Individual dots represent each participant’s median rating across the repeated measures

The children’s responses are visualised in Fig. 2. Overall, children’s naturalness ratings showed the expected directionality, such that unlicensed PSI uses were rated less natural than licensed ones (P(β < 0) = 1 for all PSIs). 11–12-year-olds do, therefore, demonstrate an awareness of the distributional restriction of these expressions. In licensing contexts, there was weak evidence for higher naturalness ratings for the (affirmative) PPI conditions compared to the (negative) NPI conditions ($\hat{\beta}$ = 0.41, CrI = [− 0.09, 0.91], P(β > 0) = 0.95), which may reflect a preference for non-negated utterances in general, or integration costs of licensed NPIs—an effect that was absent in adults. Contrary to the adult participant sample, we only found weak evidence for an interaction between the licensing status and the contrast between NPIs and PPIs ($\hat{\beta}$ = 0.44, CrI = [− 0.22, 1.10], P(β > 0) = 0.90). Instead, the model indicated differences that were specific to the comparison between *jemals*, on the one hand, and *absolut*, *durchaus*, and *so recht*, on the other. Direct comparisons between these expressions indicated that 11–12-year-olds showed a higher acceptance of unlicensed uses of *absolut*, *durchaus*, and *so recht*, than of *jemals* (P(β < 0) = 1 for all comparisons).

#### Comparison Between Groups

In order to address the question whether 11–12-year-olds differ from adults in their comprehension of PSIs, we built a model on the combined data sets. We used the same model parameters as outlined above, but additionally included the sample (adults/children) as factor interacting with the factors context and PSI. We added this factor as sum coded contrast (0.5, −0.5). The model indicated an interaction between context and group ($\hat{\beta}$ = − 0.94, CrI = [− 1.38, − 0.49], P(β < 0) = 1), such that, across all four PSIs, children made a smaller, less categorical, difference in their naturalness ratings for licensing and non-licensing contexts than adults (see also Table 3). Within the licensing contexts, children rated the sentences as less natural overall than adults ($\hat{\beta}$ = 0.61, CrI = [0.25, 0.95], P(β > 0) = 1). Additionally, we found weak evidence that children generally gave higher ratings for PSIs in non-licensing contexts than adults ($\hat{\beta}$ = -0.33, CrI = [− 0.72, 0.06], P(β < 0) = 0.95). With regard to group differences for particular PSIs, we did not find strong evidence for any such effects in the licensed PSI conditions (P(β < 0) < 0.90 or all comparisons). That is, for the grammatical target sentences, there were no PSI-specific differences between the two groups. In non-licensing contexts, on the other hand, there was evidence for group differences in the comparison between the two NPIs ($\hat{\beta}$ = 0.57, CrI = [+0.00, 1.15], P(β > 0) = 0.98) and in the comparison between the two PPIs ($\hat{\beta}$ = 0.64, CrI = [0.08, 1.18], P(β > 0) = 0.99): Both adults and children rejected unlicensed *jemals* ($\hat{\beta}$ = − 0.04, CrI = [− 0.50, 0.42], P(β > 0) = 0.43), but children showed a higher acceptance for unlicensed *so recht* than adults ($\hat{\beta}$ = 0.52, CrI = [0.05, 0.99], P(β > 0) = 0.99). With regard to the PPIs, children showed a higher acceptance for anti-licensed *durchaus* than adults ($\hat{\beta}$ = 0.72, CrI = [0.28, 1.17], P(β > 0) = 1), while both groups accepted anti-licensed *absolut* to a similar—unexpectedly high—degree ($\hat{\beta}$ = 0.11, CrI = [− 0.33, 0.56], P(β > 0) = 0.68).

**Table 3:** Adults’ and 11–12-year-olds’ mean naturalness ratings on a 1–7 Likert scale for the eight conditions of the experiment. Standard deviation in parentheses

PSI	Context	Adults	Children
*absolut* (PPI)	licensing	5.21 (1.64)	4.71 (1.81)
	anti-licensing	3.63 (1.81)	3.75 (1.66)
	Difference score (licensed—anti-licensed)	1.58 (1.14)	0.96 (1.38)
*durchaus* (PPI)	licensing	5.76 (1.40)	5.13 (1.81)
	anti-licensing	2.63 (1.59)	3.47 (1.75)
	Difference score (licensed—anti-licensed)	3.14 (1.46)	1.66 (1.86)
*jemals* (NPI)	licensing	5.27 (1.69)	4.26 (2.00)
	non-licensing	1.95 (1.37)	1.88 (1.12)
	Difference score (licensed—unlicensed)	3.32 (1.52)	2.38 (1.46)
*so recht* (NPI)	licensing	5.50 (1.50)	4.69 (1.81)
	non-licensing	3.10 (1.67)	3.778 (1.71)
	Difference score (licensed—unlicensed)	2.40 (1.22)	0.92 (1.74)

## Discussion

Using an auditory naturalness rating task, the current study investigated 11–12-year-olds’ comprehension of four German PSIs. It yielded three main results: First, we found that 11–12-year-old children trend towards adult-like comprehension of PSIs in the directionality of their responses to all four PSIs. For the NPI *jemals*, children’s responses did not differ from those of adults, indicating that they know its distributional restriction. For the NPI *so recht* and the PPI *durchaus*, on the other hand, children did not categorically reject un-/anti-licensed uses. Finally, we found that neither group consistently rejected anti-licensed uses of the PPI *absolut.* Altogether, our results thus indicate that the acquisition of PSIs takes place across a much broader period of childhood than accredited by previous studies (Lin et al. 2015, 2018; Musolini 1998; O’Leary and Crain 1994; Tieu 2013; Tieu and Lidz 2016; Xiang et al. 2006).

We have investigated a relatively old age group of 11–12-year-old children. The motivation for targeting children at this age was that crucial maturational milestones in the neurocognitive development of the language network are reported to have been reached by then (see above). Indeed, since we found that one of the investigated PSIs, *jemals*, was understood in an adult-like manner, it seems that the foundation for the comprehension of the distributional restrictions of PSIs is in place by this age and that 11–12-year-olds were able to identify and reject unlicensed uses in the sentence rating task. The remaining differences between the tested PSIs, however, require further scrutiny. In the following, we will discuss distributional differences and the special status of attenuating PSIs as potential causes for the observed differences, and will put into question the PPI-hood of *absolut*.

### Distributional Differences Between the Tested NPIs

In the corpus data above, we reported that the NPIs *so recht* and *jemals* are similarly frequent in child-directed speech, but vary with regard to their distribution over different licensing environments. We hypothesised that this may well affect the acquisition process, such that a greater variety of licensers in the input could facilitate the abstraction of the rule governing the distribution of the NPI: The different licensing contexts provide converging pieces of evidence for a common linguistic property—downward entailment, nonveridicality, or alike—underlying them all. The results from our rating study lend some support to this idea: Children’s comprehension of *jemals*, which occurred with a broader range of licensers in the corpus, was at an adult-like level, whereas the results for *so recht*, which occurred exclusively under sentential negation, indicated that 11–12-year-olds had not yet learned that it cannot occur in affirmative contexts. Future investigations will have to tell whether this contrast generalises to other languages or NPIs.

A second hypothesis that followed from the corpus data was that *so recht* is a prime candidate for a conservative widening strategy, i.e., an acquisition process wherein the dominance of particular licensers (here, sentential negation) favours an initial analysis of the NPI as being lexically dependent on said licenser. In the present case, this would lead to an analysis where the acceptability of *so recht* is dependent on its co-occurrence with *nicht* (‘not’). In later acquisition stages, this analysis would then be revised to reflect the general linguistic property licensing *so recht*. Crucially, however, at no stage in the process does this account predict that completely unlicensed uses of *so recht* would be considered acceptable by the language learner.^10^ The results from our study, wherein unlicensed *so recht* was rated much more natural by children than by adults, thus do not match with the conservative widening account. In our view, the account’s assumption that children’s initial analysis reflects a dependency relation, rather than a mere lexical collocation, between NPI and licenser may be too strong, particularly regarding its prediction that NPIs in other licensing environments should be altogether rejected by children at this stage. Instead, we will need a theory of NPI acquisition that can contend with (i) an asymmetry in production and comprehension, such that both the rarity of unlicensed NPI uses in production and the tolerance for unlicensed uses in comprehension are accounted for, and that (ii) can deal with distributional differences in NPIs, including both NPIs with a small set of relatively homogeneous licensers and NPIs with variable licensers, such as discussed on the example of *jemals* above.

### On Attenuating PSIs

The NPI *so recht* and the PPI *durchaus*, which were accepted in non-licensing contexts to a higher degree by 11–12-year-olds, both have an attenuating function. According to Israel (1996, 2011), attenuating PSIs render an assertion less informative than a contextually available alternative. With *so recht* in (13), for instance, the negation of the high degree modifier renders the assertion vague about the extent to which the speaker actually (dis-)liked the book. In (14), too, the assertion with *durchaus* carries the implicature that there are in fact aspects of the book that the speaker did not like. This is further evidenced by the oddness of discourse continuation (14a) compared to (14b).

**Table:**
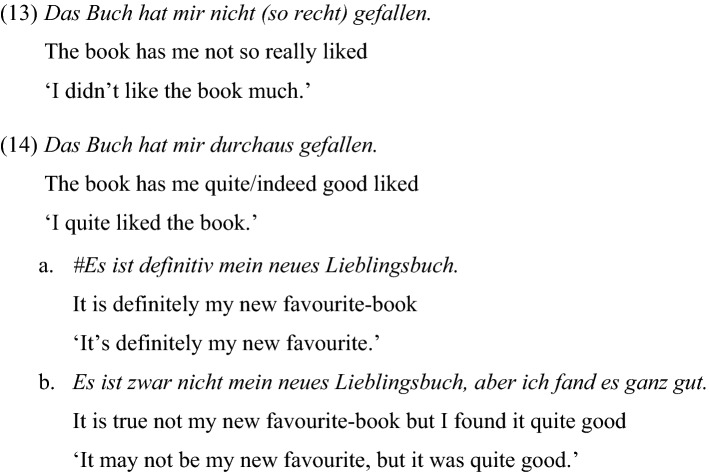

An open question is whether attenuating PSIs are potentially delayed in their acquisition compared to other PSIs. If so, this would constitute an alternative, input-independent, explanation for the contrast between *so recht* and *jemals* in the results for 11–12-year-olds. On the surface, the lexical operators that license attenuating PSIs are the same as for other PSIs. A lexical association between licensing operator and PSI should thus be equally easy to form. What may be more challenging to acquire, however, is the generalised property underlying their distribution, namely that they are restricted to contexts where the PSI makes the assertion weaker. The pragmatic power of intentionally producing a less informative sentence lies in all that is *not said*. It can be pragmatically desirable to avoid the stronger assertion if a speaker does not want to commit to it for lack of evidence (i.e., they do not know whether the stronger assertion holds), but also out of more “strategic” concerns in interpersonal communication, e.g., to be more polite (15) (Brown and Levinson 1987; regarding politeness and PSIs: Israel 2011:109ff). On the other hand, given a supporting discourse context, the attenuated assertion can sometimes also be understood as understatement, such that the speaker actually means to communicate the stronger statement but asserts the weaker one for pragmatic effect, often to be humorous (16). The complex social-pragmatic functions of attenuated assertions with PSIs may render them more difficult to acquire, particularly at early stages of children’s development when they are still known to struggle with pragmatic processes, including the drawing of implicatures (Huang and Snedeker 2009; Noveck 2001; but cf. Katsos and Bishop 2011), the comprehension of irony such as in ironic understatements (Demorest et al. 1983; Recchia et al. 2010), and the knowledge of the markers and the purpose of politeness in language (Nippold et al. 1982; Yoon 2019). We thus consider it an important avenue for further research to more closely investigate the differences between emphatic and attenuating PSIs.

**Table:** 

(15)	(*After a teacher explained something to class:*)											
	*Ich*	*habe*	*das*	*noch*	*nicht*	*(so*	*recht)*	*vertanden,*	*könnten*	*Sie*	*das*	*wiederholen?*
	I	have	this	still	not	so	really	understood	could	you	this	repeat
	‘I didn’t (quite) understand this yet, could you repeat it?’											
(16)	(*About someone who just utterly failed at a task:)*											
	*Na,*	*das*	*hat*	*ja*	*nicht*	*so*	*recht*	*geklappt.*				
	Well	this	has	yes	not	so	really	worked				
	‘Well, that didn’t work out so well.’											

### The High Tolerance for the Anti-licensed PPI *Absolut*

With regard to the tested PPIs, our results show that 11–12-year-olds are more accepting of anti-licensed uses of *durchaus* than adults, suggesting that its distributional restriction has not been fully acquired yet. Similarly, we also found a high acceptance of anti-licensed uses of *absolut*. Crucially however, the latter effect was also present in adults. That adults would assign such high naturalness ratings for anti-licensed *absolut* puts into question whether its classification as PPI in CoDII (Trawiński and Soehn 2008) is correct. In fact, we found several instances of *absolut* in the scope of negation on the web (17, 18). In all of these cases, the sentences are understood to indicate that a property holds in principle, but does not hold completely (*nicht absolut,* ‘not absolutely’). The same interpretation is available for our stimulus material. To illustrate, consider one of our items, (12f), repeated with a supporting context in (19). We thus conclude that *absolut* is not a PPI.

**Table:**
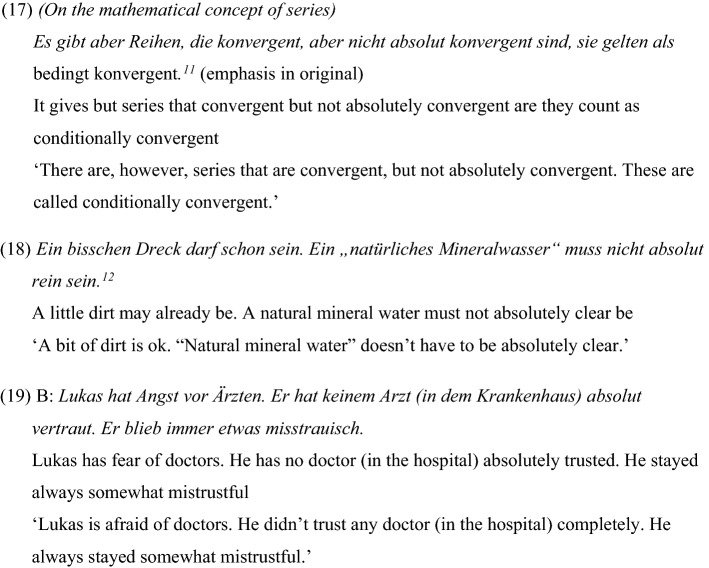

## Conclusion

Our study on the comprehension of German PSIs has found a range of differences between 11–12-year-old children and adults that indicate that the acquisition of some polarity-sensitive expressions, like *jemals*, may be completed by age 12, while the acquisition of others, like *so recht* and *durchaus*, is still ongoing. The sources of this acquisitional delay, which we have argued to be potentially attributable to differences in the language input or to differences in the status of PSIs as attenuating or emphatic, require further research. Although our study is limited in scope and therefore had to leave many questions about the acquisition of PSIs unanswered, the mere fact that this process extends at least into late childhood and possibly adolescence hopefully inspires a new line of work. Fruitful avenues that could provide more insight into this challenging phenomenon may lie in the extension of the studied age groups, particularly in filling the gap between the 5-year-olds studied before and the 11–12-year-olds studied here, but also in the application of novel experimental paradigms and methodologies available for older children (e.g., EEG, eye-tracking, sentence completion, but also assessments of pragmatic reasoning skills and of regular language input in the form of reading).

## Data Availability

All data and code associated with the experiment reported in this paper are available at the following data repository: https://osf.io/apgnv/

## Appendix

Below we list the test sentences used for the experiment. The experiment used 32 critical items that were presented in 8 conditions. These conditions can be reconstructed from the optional expressions in parentheses. Additionally, there were 16 filler items, also listed below.

## Target Items

1. Paul hat (der/kein) Kuchen von der Bäckerei (so recht/ jemals/ absolut/ durchaus) geschmeckt.
2. Anna hat (der/kein) Spielplatz in der Innenstadt (so recht/ jemals/ absolut/ durchaus) gefallen.
3. Thomas hat (der/kein) Kletterpark in der Region (so recht/ jemals/ absolut/ durchaus) gefallen.
4. Chris hat (die/keine) Antwort in dem Test (so recht/ jemals/ absolut/ durchaus) gewusst.
5. Dana hat (das/kein) Ziel von der Klassenreise (so recht/ jemals/ absolut/ durchaus) gefallen.
6. Lukas hat (dem/keinem) Arzt in dem Krankenhaus (so recht/ jemals/ absolut/ durchaus) vertraut.
7. Tina hat (das/kein) Thema in der Deutschstunde (so recht/ jemals/ absolut/ durchaus) interessiert.
8. Marc hat (die/keine) Aufgabe in der Mathearbeit (so recht/ jemals/ absolut/ durchaus) verstanden.
9. Laura hat (die/keine) Zutat für den Kuchen (so recht/ jemals/ absolut/ durchaus) gewusst.
10. Leon hat (dem/keinem) Pferd auf dem Bauernhof (so recht/ jemals/ absolut/ durchaus) getraut.
11. Bianca hat (das/kein) Geschenk von der Patentante (so recht/ jemals/ absolut/ durchaus) gefreut.
12. Julian hat (der/kein) Hund aus dem Tierheim (so recht/ jemals/ absolut/ durchaus) gehorcht.
13. Nadine hat (die/keine) Warnung vor dem Sturm (so recht/ jemals/ absolut/ durchaus) mitbekommen.
14. Florian hat (der/kein) Nachtisch von der Oma (so recht/ jemals/ absolut/ durchaus) geschmeckt.
15. Robert hat (die/keine) Regel von dem Spiel (so recht/ jemals/ absolut/ durchaus) beachtet.
16. Melanie hat (den/keinen) Ausflug in den Zoo (so recht/ jemals/ absolut/ durchaus) genossen.
17. Phillip hat (die/keine) Anweisung von dem Trainer (so recht/ jemals/ absolut/ durchaus) verstanden.
18. Lea hat (das/kein) Buch über die Dinosaurier (so recht/ jemals/ absolut/ durchaus) begeistert.
19. Nele hat (der/kein) Auftritt in der Show (so recht/ jemals/ absolut/ durchaus) interessiert.
20. Malte hat (die/keine) Mathestunde in der Schule (so recht/ jemals/ absolut/ durchaus) gemocht.
21. Merle hat (die/keine) Werbung aus dem Radio (so recht/ jemals/ absolut/ durchaus) mitbekommen.
22. Tobi hat (den/keinen) Ratschlag von den Eltern (so recht/ jemals/ absolut/ durchaus) beachtet.
23. Nina hat (das/kein) Geheimnis von der Freundin (so recht/ jemals/ absolut/ durchaus) überrascht.
24. Felix hat (die/keine) Geschichte über die Wikinger (so recht/ jemals/ absolut/ durchaus) geglaubt.
25. Sarah hat (das/kein) Beispiel für die Aufgabe (so recht/ jemals/ absolut/ durchaus) geholfen.
26. Lars hat (das/kein) Foto in dem Fotoalbum (so recht/ jemals/ absolut/ durchaus) interessiert.
27. Lina hat (den/keinen) Urlaub auf dem Bauernhof (so recht/ jemals/ absolut/ durchaus) genossen.
28. Lasse hat (das/kein) Eis im Schwimmbad (so recht/ jemals/ absolut/ durchaus) geschmeckt.
29. Sophie hat (die/keine) Aufführung im Theater (so recht/ jemals/ absolut/ durchaus) begeistert.
30. Theo hat (die/keine) Fahrt mit dem Reisebus (so recht/ jemals/ absolut/ durchaus) gemocht.
31. Lotte hat (der/keiner) Achterbahn auf dem Jahrmarkt (so recht/ jemals/ absolut/ durchaus) getraut.
32. Nils hat (die/keine) Wanderung auf der Klassenfahrt (so recht/ jemals/ absolut/ durchaus) begeistert.

## Filler Items

1. Lucy hat einen Pool zuhause, aber sie schwimmt lieber im Meer.
2. Greta hat einen Roller, aber sie fährt lieber Fahrrad.
3. Pascal hat seine Tischtennisschläger dabei, aber er spielt lieber Fußball.
4. Robin spielt gerne Fangen, aber heute spielt er lieber Verstecken.
5. Victoria spricht zuhause Deutsch, aber in der Schule spricht sie Englisch.
6. Luke kennt ein Rezept mit Nudeln, aber heute kocht er zum Mittagessen Reis.
7. Richard mag Katzen, aber er hat einen Hund.
8. Nadja backt gerne Kuchen, aber heute backt sie mit einer Freundin Kekse.
9. Peter hat den Kuchen, der viele Nüsse enthielt, sehr oft gebacken.
10. Fynn hat den Schulranzen, der mit Drachen bedruckt ist, zum Geburtstag bekommen.
11. Jennifer hat das Pony, das ein braunes Fell hat, sehr gut geputzt.
12. Emily kauft das T-Shirt, das rote Punkte hat, in dem Kaufhaus ein.
13. Tim hat sich mit dem Freund, der den größten Garten hat, verabredet.
14. Oliver sucht sich den Spielplatz, der die meisten Schaukeln hat, zum Spielen aus.
15. Niklas hat den Film, der sehr lustig war, zu Ende geguckt.
16. Pia hat die Nachtwanderung, die durch den Wald ging, sehr viel Spaß gemacht.

## Footnotes

1 For PPIs, the term anti-licensed, rather than unlicensed, is used because (1) a PPI in a sentence without negation is licensed, and only turns odd with negation that “anti-licenses” it, and (2) licensing violations for NPIs and PPIs are qualitatively different (see e.g., Szabolcsi 2004). For the same reason, we use #, indicating pragmatic oddity, instead of *, indicating ungrammaticality, in the provided examples of anti-licensed PPIs.

2 A function *f* of type < *σ,τ* > is downward entailing iff for all *x,y* of type *σ* for which *x ⇒ y: f(y) ⇒ f(x).*

3 A function *f* is subjectively nonveridical iff *f(p)* does not entail or presuppose that p is true in some individual's epistemic model M_E_(x).

4 A function *f* if anti-additive iff *f(A* ∨ *B)* ⇔ *f(A)* ∧ *f(B). Not*, for instance, is anti-additive because *f(A* ∨ *B)* = *Anna did not sing or dance* is meaning-equivalent to *f(A)* ∧ *f(B)* = *Anna did not sing and Anna did not dance.*

5 This is a broad description of the theoretical accounts underlying a class of scalar approaches. We refer the reader to the cited works for formal details.

6 NPIs, for example, can be licensed by composed expressions such as ‘*More than x but fewer than y’*, which are much less frequent than the licenser *not*. Nevertheless, adults are confident in their judgments of such expressions as licensers.

7 These were manually checked to be degree modifier uses of the phrase *so recht*. The initial search on DeReKo yielded 7.454 instances, from which we excluded 766 instances of *so recht* in syntactically unrelated structures, e.g., in *Du hast ja SO recht* (‘You are SO right about this’).

8 Audacity® software is copyright © 1999–2021 Audacity Team. The name Audacity® is a registered trademark.

9 This notation indicates the expected mean value of the estimated parameter, $\hat{\beta}$, the lower and upper bounds of the 95 percent credible interval, CrI, and the posterior probability, given the model and the data, for the hypothesis that the parameter value would be bigger than 0, P(β > 0).

10 An alternative interpretation of the high naturalness ratings for *so recht* in affirmative contexts that would be compatible with conservative widening is that 11–12-year-olds have not formed any analysis of *so recht*, yet. We consider this unlikely given that their naturalness ratings for the sentences with *so recht* are comparable to their ratings for the other PSIs. We would have expected a degradation in the ratings if the participants had not been familiar with the expression at all.

## References

[CR1] Ambridge, B. (2012). Assessing grammatical knowledge (with special reference to the graded grammaticality judgment paradigm). In E. Hoff (Ed.), *Research methods in child language: A practical guide* (pp. 113–132). Wiley-Blackwell. 10.1002/9781444344035.ch8.

[CR2] Audacity Team. (2019). *Audacity®|Free, open source, cross-platform audio software for multi-track recording and editing.* (Version 2.3.2). https://audacityteam.org/.

[CR3] Behrens H (2006). The input–output relationship in first language acquisition. Language and Cognitive Processes.

[CR4] Broce I, Bernal B, Altman N, Tremblay P, Dick AS (2015). Fiber tracking of the frontal aslant tract and subcomponents of the arcuate fasciculus in 5–8-year-olds: Relation to speech and language function. Brain and Language.

[CR5] Brown P, Levinson SC (1987). Politeness: Some universals in language usage.

[CR7] Bürkner, P. C. (2017). brms: An R package for Bayesian multilevel models using Stan. *Journal of Statistical Software*. 10.18637/jss.v080.i01

[CR6] Bürkner P-C, Vuorre M (2019). Ordinal regression models in psychology: A tutorial. Advances in Methods and Practices in Psychological Science.

[CR8] Chierchia, G. (2004). Scalar implicatures, polarity phenomena and the syntax/pragmatics interface. *Structures and Beyond*.

[CR9] Chierchia G (2013). Logic in grammar: Polarity, free choice, and intervention. Oxford University Press.

[CR10] Demorest A, Silberstein L, Gardner H, Winner E (1983). Telling it as it isn’t: Children’s understanding of figurative language. British Journal of Developmental Psychology.

[CR11] Dick F, Wulfeck B, Krupa-Kwiatkowski M, Bates E (2004). The development of complex sentence interpretation in typically developing children compared with children with specific language impairments or early unilateral focal lesions. Developmental Science.

[CR12] Drummond, A. (2013). *Ibex farm*. http:spellout.net/ibexfarm

[CR13] Giannakidou A (1998). Polarity sensitivity as (non) veridical dependency.

[CR14] Giannakidou, A. (2019). Negative and positive polarity items. In P. Portner, C. Maienborn and K. von Heusinger (Eds.), *Semantics—Sentence and information structure* (pp. 69–134). De Gruyter Mouton. 10.1515/9783110589863-003

[CR15] Gualmini A (2004). Some knowledge children don’t lack. Linguistics.

[CR16] Hahne A, Eckstein K, Friederici AD (2004). Brain signatures of syntactic and semantic processes during children’s language development. Journal of Cognitive Neuroscience.

[CR17] Huang YT, Snedeker J (2009). Semantic meaning and pragmatic interpretation in 5-year-olds: Evidence from real-time spoken language comprehension. Developmental Psychology.

[CR18] Israel M (1996). Polarity sensitivity as lexical semantics. Linguistics and Philosophy.

[CR19] Israel M (2011). The grammar of polarity: Pragmatics, Sensitivity, And The Logic Of Scales. Cambridge University Press.

[CR20] Kadmon N, Landman F (1993). Any. Linguistics and Philosophy.

[CR21] Karanth P, Suchitra MG, Scholes RJ (1993). Literacy acquisition and grammaticality judgments in children. Literacy and language analysis.

[CR22] Katsos N, Bishop DVM (2011). Pragmatic tolerance: Implications for the acquisition of informativeness and implicature. Cognition.

[CR23] Krifka M (1995). The semantics and pragmatics of polarity items. Linguistic Analysis.

[CR24] Ladusaw, W. A. (1979). Polarity sensitivity as inherent scope relations. In *PhD thesis*. University of Texas.

[CR25] Leibniz Institute for the German Language. (2020). *German reference corpus/corpora of written contemporary language 2020-I (released on 21.01.2020)*. Leibniz Institute for the German Language. www.ids-mannheim.de/DeReKo.

[CR26] Lin J, Weerman F, Zeijlstra H (2015). Emerging NPIs: The acquisition of Dutch hoeven “need”. Linguistic Review.

[CR27] Lin J, Weerman F, Zeijlstra H (2018). Acquisition of the Dutch NPI hoeven ‘need’: From lexical frames to abstract knowledge. Language Acquisition.

[CR28] Liu M, Iordǎchioaia G (2018). Current perspectives on positive polarity. Linguistics.

[CR29] Liu M, König P, Mueller JL (2019). Novel ERP evidence for processing differences between negative and positive polarity items in German. Frontiers in Psychology.

[CR30] MacWhinney, B. (2000). *The CHILDES project: Tools for analyzing talk (third edition)*. Lawrence Erlbaum Associates.

[CR31] Miller M (1979). The logic of language development in early childhood. Springer.

[CR32] Musolino, J. (1998). *Universal grammar and the acquisition of semantic knowledge: An experimental investigation into the acquisition of quantifier-negation interaction in English*. PhD thesis. University of Maryland. https://repository.upenn.edu/ircs_reports/40.

[CR33] Nippold MA, Leonard LB, Anastopoulos A (1982). Development in the use and understanding of polite forms in children. Journal of Speech and Hearing Research.

[CR34] Noveck IA (2001). When children are more logical than adults: Experimental investigations of scalar implicature. Cognition.

[CR35] Nuñez SC, Dapretto M, Katzir T, Starr A, Bramen J, Kan E, Bookheimer S, Sowell ER (2011). fMRI of syntactic processing in typically developing children: Structural correlates in the inferior frontal gyrus. Developmental Cognitive Neuroscience.

[CR36] O’Leary, C., & Crain, S. (1994). Negative polarity items (a positive result) positive polarity items (a negative result). *Boston University Conference on Language Development*.

[CR37] R Core Team. (2019). *R: A language and environment for statistical computing*. R Foundation for Statistical Computing. https://cran.r-project.org/.

[CR38] Recchia HE, Howe N, Ross HS, Alexander S (2010). Children’s understanding and production of verbal irony in family conversations. British Journal of Developmental Psychology.

[CR39] Rigol, R. (2007). *German Rigol Corpus*. CHILDES Database. 10.21415/T50S34.

[CR40] Schneider JM, Abel AD, Ogiela DA, Middleton AE, Maguire MJ (2016). Developmental differences in beta and theta power during sentence processing. Developmental Cognitive Neuroscience.

[CR41] Schneider JM, Maguire MJ (2019). Developmental differences in the neural correlates supporting semantics and syntax during sentence processing. Developmental Science.

[CR42] Skeide MA, Brauer J, Friederici AD (2014). Syntax gradually segregates from semantics in the developing brain. NeuroImage.

[CR43] Skeide MA, Brauer J, Friederici AD (2016). Brain functional and structural predictors of language performance. Cerebral Cortex.

[CR44] Skeide, M. A., & Friederici, A. D. (2016). The ontogeny of the cortical language network. In *Nature reviews neuroscience* (Vol. 17, Issue 5, pp. 323–332). 10.1038/nrn.2016.23 10.1038/nrn.2016.23 27040907

[CR45] Szabolcsi A (2004). Positive polarity: Negative polarity. Natural Language and Linguistic Theory.

[CR46] Tieu, L. (2013). *Logic and grammar in child language: How children acquire the semantics of polarity sensitivity*. Ph.D. thesis. University of Connecticut. https://opencommons.uconn.edu/dissertations/290.

[CR47] Tieu L, Lidz J (2016). NPI licensing and beyond: Children’s knowledge of the semantics of any. Language Acquisition.

[CR48] Trawiński, B., & Soehn, J. P. (2008). A multilingual database of polarity items. In *Proceedings of the 6th international conference on language resources and evaluation, LREC 2008* (pp. 273–278).

[CR49] Van Rooy R (2003). Negative polarity items in questions: Strength as relevance. Journal of Semantics.

[CR50] Vissiennon K, Friederici AD, Brauer J, Wu CY (2017). Functional organization of the language network in three- and six-year-old children. Neuropsychologia.

[CR51] von Fintel K (1999). NPI licensing, Strawson entailment, and context dependency. Journal of Semantics.

[CR52] Von Stutterheim, C. (1989). *German Caroline Corpus*. CHILDES Database. 10.21415/T5NS5S.

[CR53] Wagner KR (1985). How much do children say in a day?. Journal of Child Language.

[CR54] Wassenberg R, Hurks PPM, Hendriksen JGM, Feron FJM, Meijs CJC, Vles JSH, Jolles J (2008). Age-related improvement in complex language comprehension: Results of a cross-sectional study with 361 children aged 5 to 15. Journal of Clinical and Experimental Neuropsychology.

[CR55] Xiang, M., Conroy, A., Lidz, J., & Zukowski, A. (2006). Children’s understanding of polarity items. *Poster presented at the conference on architectures and mechanisms for language processing (AMLaP)*.

[CR56] Yoon, E. J. (2019). *Polite language reflects competing informational and social goals*. PhD thesis. Stanford University.

